# Clinical review of Toscana virus: a Mediterranean Neuroinvasive Phlebovirus

**DOI:** 10.3389/fepid.2026.1909843

**Published:** 2026-07-13

**Authors:** Ahmad Makky, Parham Elahi, Hadi Awali

**Affiliations:** 1 Department of Clinical, Surgical, Diagnostic and Pediatric Sciences, University of Pavia, Pavia, Italy; 2 Università di Parma, Parma, Italy; 3 University of Detroit Mercy, Detroit, MI, United States

**Keywords:** epidemiology, infectious diseases, phlebovirus, Toscana virus, TOSV

## Abstract

Toscana virus (TOSV) is a sandfly-transmitted phlebovirus of the family Phenuiviridae and a leading cause of summer meningitis and meningoencephalitis in the Mediterranean basin. It was first isolated in 1971 from Phlebotomus perniciosus in central Italy and circulates wherever its principal vectors are established. Seroprevalence surveys show that population exposure is common, whereas overt disease is comparatively infrequent. Among patients who become symptomatic, most present with neuroinvasive illness, which separates TOSV from related sandfly-borne phleboviruses that cause undifferentiated febrile disease. This review covers virology, pathogenesis, epidemiology, vector biology, clinical features, diagnosis, and management of TOSV infection, and it keeps a clear separation between established clinical knowledge and experimental research. The practical message has not changed in a decade: management is supportive, no licensed antiviral or vaccine exists, and the agents that attract research interest, including repurposed nucleoside analogues, venom-derived peptides, RNA-interference strategies, and candidate vaccines, remain preclinical. Given warmer Mediterranean summers and a documented rise in recognised cases, the most useful priorities are better clinician awareness, wider access to TOSV-specific molecular testing, and integrated human and entomological surveillance.

## Introduction

Toscana virus is a negative-sense, single-stranded RNA virus with a tripartite genome (1). The large (L) segment encodes the RNA-dependent RNA polymerase, the medium (M) segment encodes the glycoprotein precursor that is processed into the envelope glycoproteins Gn and Gc responsible for attachment and membrane fusion, together with a nonstructural NSm protein whose function in TOSV is not well characterised, and the small (S) segment encodes the nucleoprotein together with the non-structural NSs protein, an antagonist of the type I interferon response (1, 2). The virus was recovered in 1971 from sandflies collected in Tuscany, from which it takes its name, and was later recognised as a neurotropic human pathogen rather than only an agent of self-limited sandfly fever (3).

The epidemiology of TOSV shows a marked gap between exposure and disease. Seroprevalence in endemic regions usually falls within a broad band of roughly 6–25 percent depending on geography, age, and occupational exposure, yet only a small fraction of infected people develop clinical illness (4, 5). When disease occurs it is mainly neuroinvasive, and in central Italy the virus has long been the most frequent cause of aseptic meningitis between late spring and autumn, well ahead of the enteroviruses during the warm season (3, 6). Reports from Italy describe a recent rise in recognised neuroinvasive disease, attributed both to greater diagnostic awareness and to ecological change, although the lack of uniform mandatory surveillance across the endemic zone makes secular trends hard to quantify (7, 8).

This review sets out to answer three questions: what constitutes the established clinical and epidemiological picture of TOSV neuroinvasive disease, as distinct from research that remains preclinical; how much of the recent rise in recognised cases reflects true change in transmission rather than improved ascertainment; and which interventions carry the greatest near-term value given that no licensed therapy exists. No specific therapy is available, care is supportive, and the experimental antivirals occasionally used off-label have no efficacy data in TOSV infection. Emerging peptide therapeutics, RNA-interference approaches, and vaccine candidates are discussed as research directions, not as options that should be offered to patients (9, 10).

## Methods

This is a narrative review. We searched PubMed/MEDLINE (title/abstract and MeSH fields), Scopus and Web of Science (Title-Abstract-Keyword) from inception to March 2026. The core search string was (“Toscana virus” OR “TOSV” OR “sandfly fever”) AND (“Phlebotomus” OR “aseptic meningitis” OR “meningoencephalitis” OR “neuroinvasive” OR “epidemiology” OR “diagnosis” OR “treatment” OR “vaccine”), adapted to the syntax of each database. The PubMed search returned 489 records, the Scopus search 2,517 records, and WoS returned 1,557 records; after de-duplication, 2151 titles and abstracts were screened and 284 full texts were assessed. Reference lists of retrieved articles and relevant reviews were hand-searched for additional sources, and surveillance reports from the European Centre for Disease Prevention and Control were consulted for epidemiological context. Priority was given to primary clinical, virological, and entomological studies, with preference for the most recent and methodologically robust evidence; experimental and preclinical work is identified as such throughout and is kept separate from established clinical practice. Full-text review included English-, French-, Spanish-, and Italian-language publications. Although the Italian and Spanish primary literature on TOSV is substantial, much of it is reachable through Scopus with English-language abstracts, and key non-English papers were translated for assessment, so residual language bias is reduced but cannot be fully excluded. Study selection and synthesis were performed by the authors.

## Virology and pathogenesis

### Genome organisation and the molecular basis of neurotropism

The TOSV genome totals roughly 12 kilobases across its three segments (1). The L segment encodes the polymerase that drives viral transcription and replication. The M segment encodes a glycoprotein precursor that is processed into Gn and Gc, which mediate attachment to the sandfly midgut and entry into mammalian cells, and the same precursor yields a nonstructural NSm protein; as in related phleboviruses, the role of TOSV NSm is incompletely defined (1, 11). The S segment, transcribed by the ambisense strategy typical of phleboviruses, encodes the nucleoprotein that encapsulates the genomic RNA and the NSs protein that subverts innate immunity (2, 12).

NSs is the main virulence determinant. By interfering with the cytosolic RNA sensors that would normally trigger interferon induction, it suppresses the early type I interferon response and allows the high-titre viraemia needed for dissemination (2, 12). The clearest experimental support for this role comes from reverse-genetics work on a lineage B strain: a recombinant virus engineered to lack NSs expression could not suppress interferon-beta induction and grew poorly in interferon-competent cells, whereas the parental virus replicated efficiently (2).

Three genetic lineages, designated A, B, and C, are distinguished by nucleotide divergence and broad geographic clustering (1). Lineage A predominates in Italy and Spain, lineage B is described in France, North Africa, and Turkey, and lineage C has never been recovered as an infectious isolate. It is defined solely by partial nucleoprotein-gene sequences amplified directly from specimens, first from the cerebrospinal fluid of a meningitis patient in Croatia and subsequently from a patient in Greece, and later from *Phlebotomus neglectus* sandflies in Croatia, with attempted cell-culture isolation unsuccessful. Because only partial sequence data exist and no replication-competent virus is available, its replicative and pathogenic phenotype cannot be characterised and its biological significance remains uncertain (1, 5, 13). The genetic diversity of the surface glycoproteins is not only a phylogenetic feature: reverse-genetics comparisons of lineage A and B strains show that the Gn and Gc sequences govern differences in replicative fitness, entry kinetics, and the infectivity of newly produced virions (14). Phenotypic studies in human neural cells and brain organoids likewise point to measurable differences between strains associated with self-limited and more persistent infection, although these *in vitro* phenotypes cannot yet be translated into reliable predictors of human disease severity (15).

### Dissemination and central nervous system invasion

After inoculation through the bite of an infected sandfly, the virus disseminates through lymphatic and haematogenous routes and becomes detectable in blood within a few days, and symptomatic patients have demonstrable viraemia for roughly the first week of illness (16). How the virus reaches the central nervous system is not fully resolved. Two non-exclusive mechanisms are proposed: direct passage across the blood-brain barrier through infected endothelium, and a Trojan horse route in which infected mononuclear cells traffic into the meninges and parenchyma. The prominence of monocytes and macrophages in the cerebrospinal fluid of some patients lends support to the leukocyte-trafficking hypothesis without excluding the alternative.

The clinical course can be described in three overlapping phases that map onto the underlying pathology. An initial viraemic phase produces fever and myalgia with little or no central nervous system involvement (16). A meningitic phase follows, with viral replication in the meninges and choroid plexus, lymphocytic inflammation, and the usual symptoms of headache, neck stiffness, and photophobia. In a minority of patients an encephalitic phase follows, with perivascular lymphocytic cuffing, microglial activation, and neuronal injury (17). The small number of fatal cases that have come to autopsy show perivascular inflammation with a predilection for the temporal lobes and brainstem, neuronal necrosis and apoptosis, and microglial proliferation, while extracranial involvement is rare.

### Viral persistence and its relationship to sequelae

Viral RNA can persist in the cerebrospinal fluid for several weeks beyond symptom onset in a subset of patients, more often those with prolonged or severe disease, even though infectious virus is generally no longer recoverable (15). The mechanism is unknown, and abortive replication in central nervous system macrophages, sequestration of RNA in extracellular vesicles, and unproven latency have all been suggested. The relationship between persistent RNA and long-term neurological sequelae is correlative, not causal. Persistence is neither necessary nor sufficient for sequelae, which points to a multifactorial process in which neuroinflammation, neuronal apoptosis, and possibly post-infectious immune mechanisms contribute alongside or independently of any residual viral material.

### Epidemiology

TOSV circulates throughout the countries bordering the Mediterranean, and its distribution follows the range of competent Phlebotomus vectors (5, 18). The quality and density of surveillance vary widely between countries, so apparent differences in burden partly reflect differences in case ascertainment rather than true differences in transmission (3).

Italy is the best-documented setting and the historical epicenter. In central Italy the virus has long been the leading identified cause of aseptic meningitis during the warm months, a finding rooted in studies from the 1980s and 1990s that placed TOSV ahead of the enteroviruses as the dominant viral cause of summer meningitis with an identifiable cause (3, 6). National surveillance, helped by the fact that neuroinvasive TOSV infection has been notifiable in Italy since 2016, has more recently quantified a clear rise, with incidence in 2022 and 2023 about 2.6 times higher than in 2016–2021, coinciding with anomalously warm seasons and increased activity of other vector-borne diseases (7). The same data identified working-age adults, older men, and residents of rural municipalities as groups warranting particular attention for preventive messaging (7). Year-to-year counts remain volatile because transmission is sensitive to seasonal vector abundance, so a single epidemic year should not be read as a stable trajectory (8).

Elsewhere in the northern Mediterranean the burden is confirmed but lower or less completely documented. In southern France the virus is confined to Mediterranean coastal regions including Provence and Corsica, where seroprevalence is appreciable and case series have established it as a recognised cause of summer neuroinvasive disease, yet the documented case count stays modest relative to Italy for reasons that probably combine lower incidence with under-diagnosis and reporting bias (19, 20). In Spain, molecular evidence of TOSV in P. perniciosus and P. perfiliewi is widespread along the Mediterranean coast, seroprevalence reaches the upper end of the regional range in some coastal populations, and neuroinvasive cases have been documented mainly in Andalusia and Catalonia, again against less systematic surveillance than in Italy (21–23). In Turkey, serological and molecular evidence places the virus across several regions of Anatolia, with documented neuroinvasive cases and evidence of exposure among blood donors (24).

Two patterns warrant attention. The first is the Greece and Cyprus discrepancy, in which serosurveys show substantial historical exposure yet confirmed neuroinvasive cases are scarce or absent in the published record. This is better read as a problem of serological surveillance than as a true biological paradox. The high Cypriot figures derive from assays run against several sandfly fever viruses, and binding-antibody tests cross-react among the co-circulating Naples, Sicilian, and Toscana viruses, so apparent TOSV exposure is readily overestimated where virus-specific neutralisation is not performed. Vector composition compounds this, since *Phlebotomus papatasi*, the principal vector of Sandfly fever Naples virus and not a competent vector of TOSV, predominates in parts of the eastern Mediterranean, so a high anti-phlebovirus seroprevalence there need not indicate TOSV circulation (25). Whether any genuine excess of unrecognised neuroinvasive disease remains once cross-reactivity is accounted for, and what role host factors or a less neurovirulent lineage might play, cannot be resolved without neutralisation-based serosurveys and systematic molecular testing. The second is North Africa, where serological and entomological evidence from Tunisia and Algeria confirms circulation but published clinical case data are sparse, and where serological interpretation is complicated by co-circulating related phleboviruses that can cross-react in antibody assays (26, 27).

**Figure 1:**
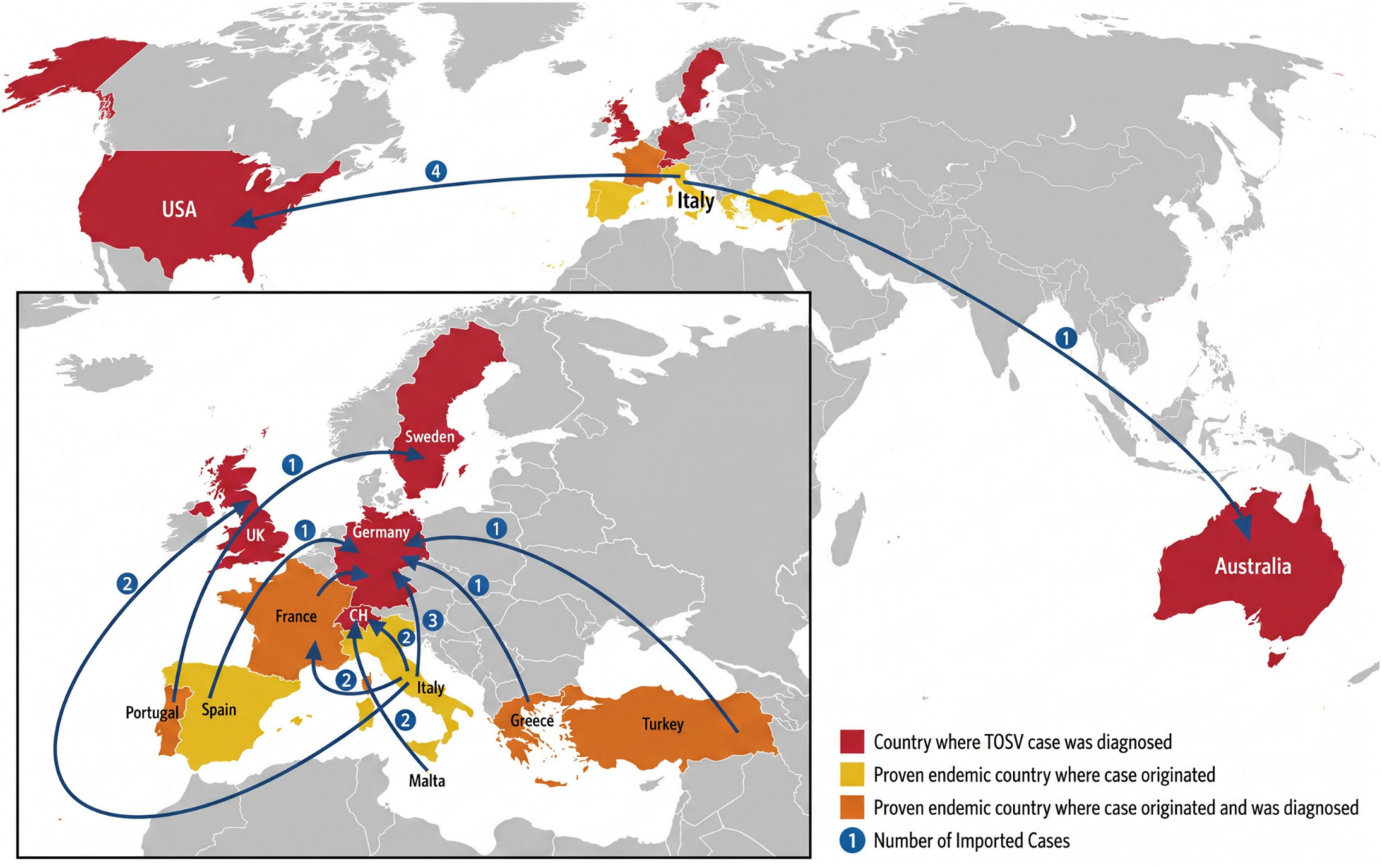
Geographical distribution of toscana virus infections. Schematic map of documented TOSV importation by travellers returning from the endemic Mediterranean basin, with a magnified inset of the European endemic zone. (30) Arrows run from the country of presumed acquisition to the country of diagnosis, with the number indicating imported cases reported for that route. These represent importation during the incubation period, not autochthonous transmission; no locally acquired TOSV outside the established endemic range has been documented. Created with BioRender.

Beyond the established endemic zone, sporadic infections diagnosed in northern European travellers returning from Mediterranean holidays have been recognised for decades and reflect importation during the incubation period rather than local transmission (Figure 1) (28, 29). Ecological modelling anticipates a meaningful northward expansion of suitable Phlebotomus habitat over the coming decades under moderate-to-high emissions scenarios, potentially extending suitable conditions into parts of central Europe (10). Autochthonous TOSV transmission in central Europe has not been documented. Establishment would require competent vector populations at sufficient density, suitable vertebrate hosts, a long enough warm season for viral replication within the vector, and human behaviours that bring people into contact with biting sandflies, conditions that have not yet aligned beyond the current range (10).

### Vector biology and transmission

The confirmed competent vectors of TOSV are Phlebotomus perniciosus, predominant in the northern Mediterranean, and P. perfiliewi, more important in central and southern areas (1, 5). Competence rests on virus isolation from wild-caught females, experimental demonstration of replication, and successful transmission (30). A persistent source of confusion should be corrected, since P. papatasi, although abundant across the Mediterranean and the Middle East, is the principal vector of the related Sandfly fever Naples virus and is not an established vector of TOSV (1). The historical conflation of these viruses under overlapping clinical labels has been resolved by modern molecular characterisation, and the two should be kept distinct (18).

P. perniciosus occupies rural, peri-urban, and occasionally urban habitats across southern Europe, North Africa, and parts of the Middle East, breeding in organic-rich soils, animal shelters, and crevices (5). It is active through the warmer half of the year with a midsummer-to-early-autumn peak, feeds on both humans and a range of animals, and is short-lived, surviving only a few weeks, which limits its capacity as a vector (30).

### Intra-vector dynamics and transmission parameters

**Figure 2:**
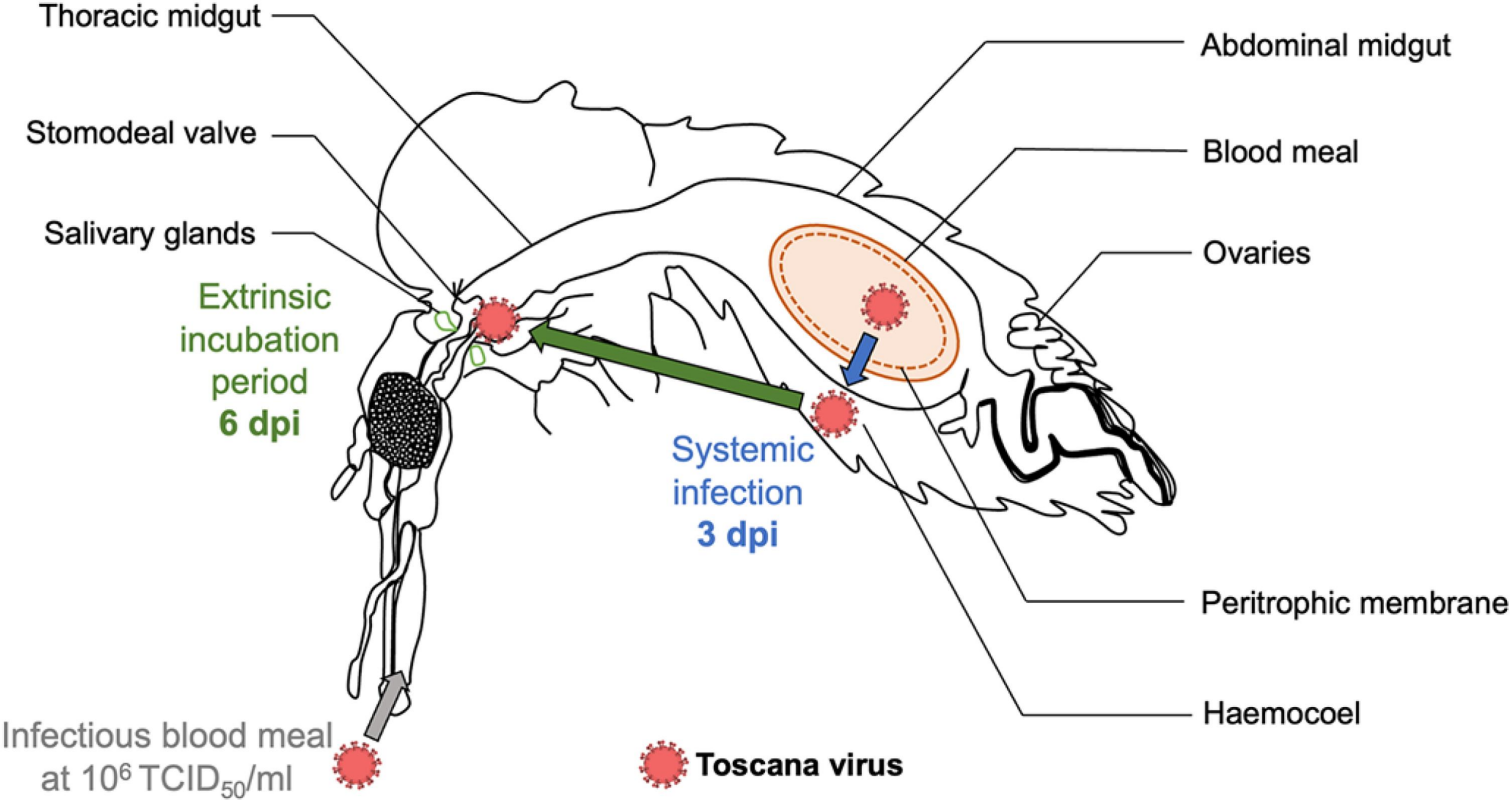
Intra-vector dynamics of toscana virus in *phlebotomus perniciosus*. Schematic of sandfly digestive and reproductive anatomy showing the course of TOSV infection after an infectious blood meal (10⁶ TCID₅₀/mL). The virus replicates in the midgut and disseminates through the haemocoel, producing systemic infection by 3 days post-infection, then reaches the salivary glands, with the extrinsic incubation period estimated at 6 days. (30).

The transmission cycle follows the general arboviral pattern. A female ingests a viraemic blood meal, the virus replicates in the midgut, disseminates through the haemocoel to secondary tissues including the salivary glands, and is then transmitted in saliva at a later blood meal (Figure 2) (30). Much of what is known quantitatively about this cycle rests on a single experimental study, so the following parameters should be read as provisional pending independent replication. Experimental infection of P. perniciosus has given the first direct insight into this cycle, with systemic dissemination detectable within about three days of an infectious blood meal and the extrinsic incubation period, the interval from acquisition to transmissibility, estimated at around six days (30). Given this interval is much shorter than the vector's lifespan, the extrinsic incubation period is unlikely on present evidence to be the factor that limits transmission (30). The same work also found that infection can alter vector life-history traits, including the timing of egg hatching, which could delay the emergence of infected sandflies with uncertain consequences for transmission (30). Transmission efficiency depends on the titre of the ingested blood meal and on ambient temperature, which together govern the probability and speed of dissemination, with replication favoured across a moderate temperature window and impaired at the extremes (30). These vector-level parameters plausibly connect to the recent rise in recognised Italian cases during warmer seasons: a shorter extrinsic incubation period, a longer and more intense period of vector activity, and faster larval development would each raise the number of infectious bites per season. The single available experiment was not designed to quantify the response of the extrinsic incubation period to the incremental 1–2 degree C increases observed in recent years, so the precise temperature sensitivity of TOSV transmission in *P. perniciosus* remains undefined and is a priority for temperature-controlled vector-competence studies. The incubation period in humans has also been estimated from imported and well-defined cases, supporting the short intervals seen in the vector (31).

Several alternative routes of viral maintenance have been described, all of limited epidemiological weight. Vertical, or transovarial, transmission occurs at low frequency in experimentally infected females and may help the virus persist between transmission seasons, but it cannot sustain human-relevant transmission without viraemic vertebrate hosts (32). Detection of viral RNA in male sandflies and occasional transfer to naive females raises the possibility of venereal or related maintenance routes, again relevant to overwintering rather than to human exposure (32). The detection of viral RNA in plant-sugar sources fed on by sandflies is an interesting observation from experimental work, but evidence that contaminated nectar mediates fly-to-fly transmission is anecdotal, and any such route has no demonstrated clinical relevance (30).

## Clinical manifestations

### The asymptomatic majority and mild febrile disease

The high seroprevalence in endemic populations against the relative infrequency of disease implies that most infections are asymptomatic or subclinical (4, 5). Seroprevalence in endemic regions spans roughly 6–25 percent depending on geography, age, and exposure, yet only a small minority of those infected develop overt disease; in pooled clinical data the median age of recognised cases is about 44 years and men outnumber women roughly two to one (33). Among those who become unwell, a proportion have only a brief, self-limited febrile illness, the original sandfly fever phenotype, with fever, malaise, myalgia, and arthralgia lasting a few days and without headache, meningism, or altered mentation (34). Cerebrospinal fluid is rarely sampled in such patients and, when it is, tends to be normal or to show only minimal pleocytosis.

### Meningitis

Neuroinvasive disease, and meningitis in particular, dominates the symptomatic spectrum (3). In the largest pooled series, drawn from 95 published studies and 644 neuroinvasive cases with a documented syndrome, 519 (81 percent) presented as meningitis or aseptic meningitis and 111 as encephalitis or meningoencephalitis, with myelitis rare (33). The illness usually moves through a short prodrome of abrupt fever and myalgia without meningeal signs into a meningitic phase over the following days, marked by intense, often frontal or occipital headache, photophobia and phonophobia, neck stiffness, and, in a substantial minority, positive Kernig or Brudzinski signs, often with nausea and vomiting (17). In most patients the meningeal signs and fever resolve over the next one to two weeks, although headache may linger for several weeks after the acute illness has settled, while a smaller group have a more protracted course. Atypical presentations include a purely febrile illness without meningism, meningitis with little fever in immunocompromised hosts, and a more subacute evolution over two to three weeks.

### Encephalitis

A minority of symptomatic infections involve the brain parenchyma, and encephalitis often coexists with meningitis as a meningoencephalitis rather than occurring alone (17). The features that separate parenchymal involvement from meningitis alone are altered mental status ranging from disorientation to depressed consciousness, behavioural change, seizures that are usually generalised but sometimes focal and occasionally progress to status epilepticus, and focal neurological deficits such as hemiparesis, aphasia, visual field loss, or cranial nerve palsies, with tremor or myoclonus in some cases (17). Deep coma is uncommon, and although deaths are documented the case-fatality rate with modern supportive care is well below one percent (17). In the largest pooled series the case-fatality rate was 0.43 percent, concentrated in patients over 65 with comorbidities such as diabetes and hypertension (33). The cerebrospinal fluid in encephalitis tends to show more pronounced lymphocytic pleocytosis and higher protein than in uncomplicated meningitis, while glucose stays normal or only modestly reduced.

### Uncommon presentations

Less frequent manifestations include myelitis, with limb weakness, a sensory level, and sphincter disturbance, sometimes with spinal cord signal change on imaging; a Guillain-Barre-like syndrome reported as a post-infectious or concurrent process whose causal link to TOSV is not firmly established; and radicular or cranial nerve involvement producing focal palsies. These are rare and should prompt careful exclusion of alternative diagnoses.

## Diagnosis

### Cerebrospinal fluid analysis

The cerebrospinal fluid in TOSV meningitis shows the profile expected of a viral process, namely a lymphocytic pleocytosis usually in the low hundreds of cells per microlitre that may be neutrophil-predominant only in the first day before shifting to a lymphocytic pattern, a mildly to moderately raised protein, and a glucose that is normal or only slightly low, with the cerebrospinal-fluid-to-serum glucose ratio near the normal range (17). A concurrent serum glucose is needed for interpretation. This combination helps separate TOSV from bacterial meningitis, in which glucose is characteristically and substantially depressed and the pleocytosis neutrophilic, but it does not separate TOSV from other viral causes such as herpes simplex, enterovirus, or varicella-zoster virus. Gram stain and bacterial culture are negative in TOSV infection.

### Molecular and serological confirmation

Specific diagnosis rests on molecular and serological methods. Reverse-transcription PCR on cerebrospinal fluid gives the highest yield during the first week of illness, with sensitivity falling as viraemia and central nervous system viral load decline, and serum can be tested but is less sensitive (16). Well-designed TOSV-specific assays are highly specific and do not cross-react meaningfully with related phleboviruses, and positive results are best confirmed by a second method such as sequencing, with primer and probe design that accommodates lineage diversity (1). Serology complements molecular testing, particularly later in the illness when viral RNA may no longer be detectable. Specific IgM becomes detectable within the first week and persists for some months, while IgG appears slightly later and remains for years, which makes it the basis of seroprevalence studies (4, 5). Because IgM can lag the onset of symptoms, a practical approach tests an acute-phase serum and, if needed, a convalescent sample, with seroconversion or a significant rise in titre confirming recent infection, and cross-reactivity with related phleboviruses should be kept in mind, especially in North African populations where such viruses co-circulate (26, 27).

Robust head-to-head performance estimates for TOSV-specific assays are limited and derive mainly from reference-laboratory series, so test choice is best guided by the kinetics of viraemia and antibody. A staged approach follows from these kinetics: in the first week after symptom onset, cerebrospinal fluid RT-PCR is the test of choice and serum a lower-yield adjunct; from the end of the first week, as PCR yield declines, paired acute and convalescent serology becomes the mainstay, with seroconversion or a significant rise in IgM or IgG titre confirming recent infection. Where both are feasible, simultaneous early CSF PCR and baseline serology maximise the chance of confirmation across the transition between the two windows.

### Multiplex panels and neuroimaging

Multiplex molecular panels for meningitis and encephalitis allow simultaneous testing of cerebrospinal fluid for a range of pathogens within a few hours and support rational antimicrobial stewardship by allowing early discontinuation of empirical therapy when appropriate. Their drawbacks are cost and limited availability outside reference and tertiary centres, and the extent to which TOSV is interrogated depends on the panel and on the clinician's index of suspicion (10). Neuroimaging is adjunctive. Computed tomography is used to exclude mass lesions, haemorrhage, or hydrocephalus when there is altered consciousness, seizures, or signs of raised intracranial pressure, and is usually normal or non-specific in TOSV disease. Magnetic resonance imaging may show T2 or FLAIR signal change in limbic and temporal structures in a proportion of encephalitis cases and meningeal enhancement in meningiteal disease, but the appearances are non-specific, and the main value of imaging is to exclude alternatives such as herpes simplex encephalitis, which characteristically produces anterior temporal involvement with a more necrotising pattern.

### Outcomes and long-term sequelae

The prognosis of TOSV neuroinvasive disease is generally good, with full recovery in most patients and a case-fatality rate below one percent with contemporary supportive care (17). Morbidity is not negligible. A minority of survivors have clinically significant sequelae, the most common being persistent headache, followed by neurocognitive complaints such as impaired memory, concentration, and processing speed, and less often sensorineural hearing loss, post-encephalitic epilepsy, residual motor deficits, and neuropsychiatric symptoms including mood disturbance. The reported timelines vary, with some sequelae emerging during convalescence and others persisting for months or years.

Several risk factors for poorer outcome recur across case series, including older age, parenchymal involvement rather than meningitis alone, more intense cerebrospinal fluid pleocytosis, delayed diagnosis, and immunosuppression, although the supporting data are largely observational and limited by heterogeneous cohorts. The mechanisms behind sequelae are incompletely understood and partly hypothetical, and include cytokine-mediated neuroinflammation, neuronal apoptosis, the uncertain contribution of persistent viral RNA, and speculative post-infectious autoimmune processes (15). The determinants of long-term neurological outcome after TOSV infection are not well characterised and are an important target for prospective study.

## Management

### Supportive care is the standard of care

No licensed antiviral therapy exists for TOSV, and management is supportive throughout, matched to disease severity (10). This position reflects consensus practice for viral meningitis and encephalitis rather than TOSV-specific trials, which have not been done.

Mild, non-neuroinvasive febrile illness without meningeal signs can be managed as an outpatient with hydration, antipyresis and analgesia using paracetamol or non-steroidal anti-inflammatory drugs, rest, and short-interval clinical review (34). Admission is appropriate when fever is high and refractory, when vomiting prevents oral intake, when symptoms are severe, or in older patients and those with comorbidity or unreliable follow-up.

Meningitis warrants admission. The initial workup supports the diagnosis and excludes bacterial and herpetic causes, and usually includes blood cultures, full blood count and biochemistry, paired serum and cerebrospinal fluid glucose, lumbar puncture with cell count, protein, glucose, Gram stain, bacterial culture, and viral PCR including TOSV-specific testing where available, baseline and convalescent serology, and cross-sectional imaging or electroencephalography where altered consciousness or seizures are present. Supportive care centres on maintaining euvolaemia with isotonic fluids while avoiding hypotonic solutions and the risk of hyponatraemia, careful electrolyte monitoring with cautious correction of any hyponatraemia, antipyresis, effective analgesia for what is often a severe headache, antiemetics, and prompt treatment of seizures with benzodiazepines followed by an antiepileptic agent if seizures recur. Empirical antibacterial therapy and aciclovir are often started before TOSV is confirmed and should be reviewed and stopped once bacterial and herpetic causes are excluded.

Severe encephalitis needs intensive care. Airway protection and mechanical ventilation are indicated for a depressed conscious level or refractory seizures, continuous electroencephalographic monitoring is used where status epilepticus is suspected, osmotic therapy is reserved for signs of cerebral oedema or raised intracranial pressure, and neurosurgical input is sought if imaging suggests herniation. With this level of support, recovery is the usual outcome even in severe disease, and mortality stays low (17).

### Interventions with weak or no supporting evidence

Corticosteroids are not recommended for routine TOSV meningitis. The rationale for their use is extrapolated from bacterial meningitis and is not supported by TOSV-specific data, and there are theoretical harms in a viral infection, including impaired antiviral immunity and an increased risk of secondary bacterial infection. Their use might be considered only in severe encephalitis with radiographic evidence of significant cerebral oedema, and then only as an individualised decision that acknowledges the uncertain balance of benefit and risk.

Intravenous immunoglobulin is also not recommended. The supporting literature consists of isolated case reports describing apparent improvement in severe, prolonged encephalitis, from which causation cannot be inferred given the possibility of spontaneous recovery. It might be considered only in exceptional, refractory cases after a frank discussion of unproven benefit and substantial cost.

### Experimental antivirals lack human efficacy data

Two repurposed polymerase inhibitors are sometimes raised in discussion. Ribavirin inhibits the viral polymerase and reduces TOSV replication in cell culture, but it has no demonstrated clinical efficacy in TOSV infection, the human experience is limited to a handful of off-label reports in severe disease from which no conclusion about benefit can be drawn, and the drug carries appreciable toxicity, including haemolytic anaemia, teratogenicity, and hepatic and pancreatic effects. Favipiravir, another polymerase inhibitor with activity against several RNA viruses and some *in vitro* activity against related bunyaviruses, has no published human or TOSV-specific *in vitro* efficacy data, and any inference of activity is extrapolation. Neither agent is recommended for TOSV disease. At most, in a patient with rapidly progressive, life-threatening encephalitis failing maximal supportive care, such agents might be considered only under formal institutional and ethical oversight with fully informed consent, recognising that benefit is unproven and toxicity is real.

### Investigational and future therapeutic directions

**Figure 3:**
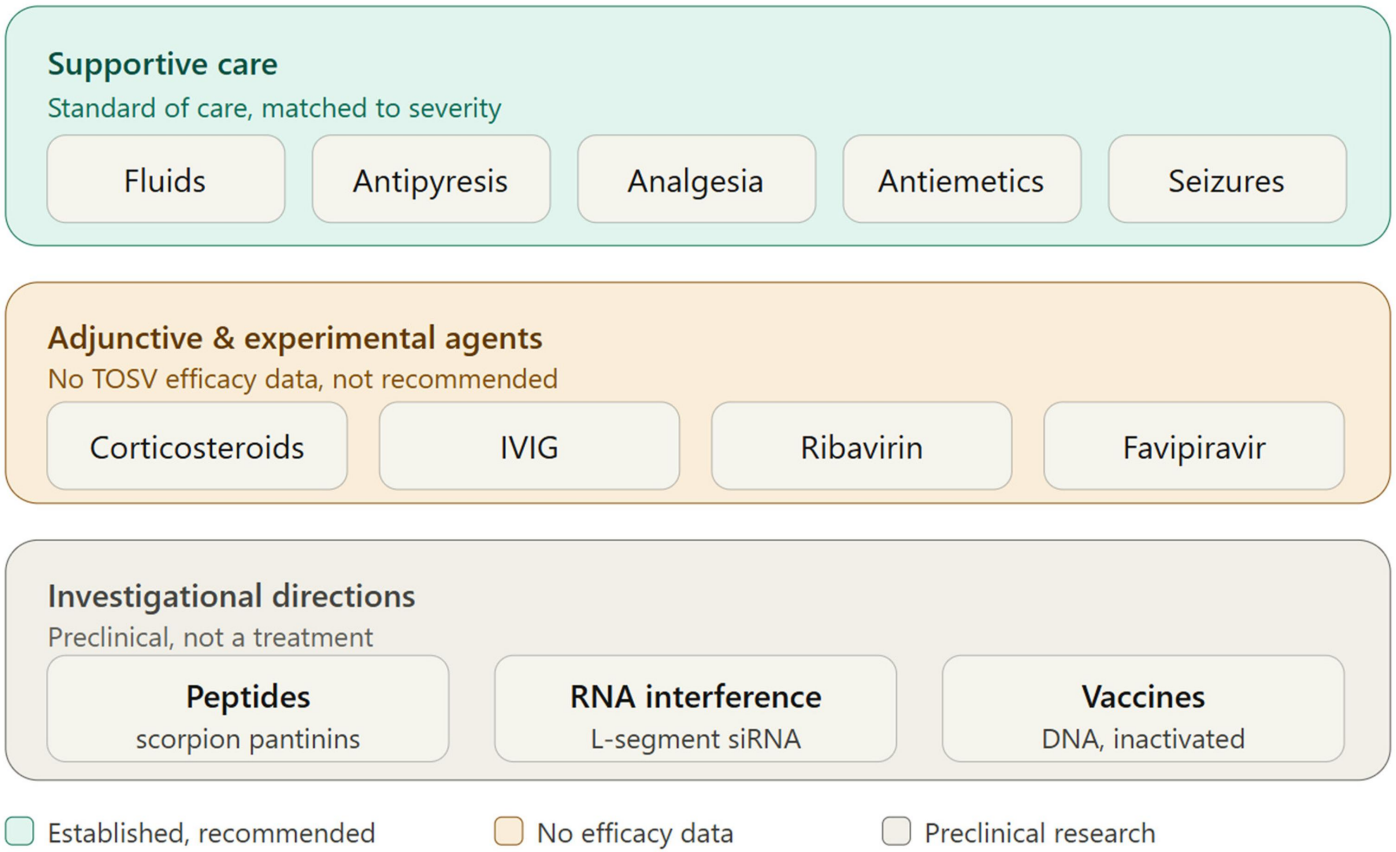
Management of toscana virus infection, organised by strength of evidence. Approaches are arranged in descending tiers of supporting evidence. Supportive care, matched to disease severity, is the only established standard of care: maintenance of euvolemia and electrolyte balance, antipyresis, analgesia, antiemetics, and seizure control, with hospitalisation for meningitis and intensive care for severe encephalitis. Adjunctive and experimental agents (corticosteroids, IVIG, ribavirin, favipiravir) have no TOSV-specific efficacy data and are not recommended; their use is confined to exceptional, individualised circumstances under appropriate oversight. Investigational directions (venom-derived peptides, RNA-interference, and candidate vaccines) are preclinical and are not treatments. No licensed antiviral or vaccine exists. Created with BioRender.

Several lines of preclinical research are sometimes cited as grounds for optimism, but each is early-stage, none has human efficacy or safety data, and realistic timelines to any clinical application are long (Figure 3). These directions should be communicated to patients and the public as research, not as treatment.

Venom-derived host-defence peptides have drawn attention after the finding that scorpion-derived pantinin peptides show antiviral activity against TOSV and the related Schmallenberg virus in cell culture, with activity against several cell types and a favourable selectivity window *in vitro* (9). The findings are restricted to cell culture, there are no *in vivo* efficacy data, the neuronal cell line used is a tumour line of limited fidelity to natural central nervous system infection, and there are no pharmacokinetic, toxicological, or blood-brain-barrier penetration data (9). Translation, if it occurs, would require animal efficacy and safety studies, regulatory submission, and phased clinical trials over several years.

RNA-interference strategies, in which short interfering RNAs target conserved viral sequences to trigger degradation of viral RNA, have shown reductions in viral replication in cell systems and in some animal experiments. The obstacles to clinical use are large, since delivery to the central nervous system is invasive, the short half-life of small interfering RNAs in cerebrospinal fluid would require repeated dosing, off-target effects remain a concern, and costs are high. No human safety data in TOSV disease exist, and the timeline to any application is long.

Vaccine development remains preclinical. Candidate approaches include DNA plasmid constructs encoding the Gn and Gc glycoproteins, which have induced neutralising antibodies in animal models, and inactivated whole-virus preparations, which have given partial protection against lethal challenge in animals. Neither has human immunogenicity, safety, or efficacy data, and questions of dosing, durability of protection, cross-lineage coverage, and manufacturing remain open. Given the usual arc of vaccine development through immunogenicity studies, process development, and phased clinical and efficacy trials, a licensed TOSV vaccine is not a near-term prospect (10).

### Diagnostic and surveillance challenges

The practical barriers to managing TOSV disease are less about therapeutics than about recognition and detection. Clinically, TOSV meningoencephalitis is indistinguishable from other viral causes, so empirical antiviral cover is the norm until specific causes are excluded, and in the Mediterranean the differential diagnosis of summer neuroinvasive disease should include TOSV so that specific testing is requested (3, 7). Laboratory capacity is uneven, since TOSV-specific PCR is concentrated in reference and specialised virology laboratories, primary and secondary hospitals often lack the capability, and the need to ship specimens introduces diagnostic delay (10). Multiplex panels that would shorten this loop are available in only a minority of hospitals across the endemic region and are limited by cost. There is a clear case for developing and deploying rapid, lower-cost point-of-care multiplex assays in endemic areas to allow real-time identification and rational antimicrobial use.

Surveillance is also incomplete. Passive reporting captures only a fraction of clinically apparent infections, with substantial under-ascertainment in primary care, in hospitals without diagnostic capability, in countries without mandatory reporting, and, by definition, among asymptomatic infections (7, 10). The true incidence of TOSV neuroinvasive disease across the Mediterranean is therefore almost certainly higher than reported figures suggest. Stronger surveillance, through notifiability during the transmission season, integration of human case reporting with entomological monitoring, and predefined thresholds that trigger enhanced testing during summer surges, would improve both situational awareness and the evidence base for resource allocation (7).

The prospect of climate-driven range expansion argues for anticipatory vector surveillance beyond the current endemic limits. Although suitable habitat is projected to extend northward over the coming decades, autochthonous transmission in central Europe has not been observed, and whether local Phlebotomus populations at the expanding margin are competent for TOSV is unknown (10). Prospective entomological surveillance in central and eastern Europe, vector-competence studies using established Mediterranean lineages, and inclusion of TOSV testing in summer meningitis workups in border regions would help detect any shift from imported to locally acquired disease early (10, 30).

## Conclusion

Toscana virus is a major and probably under-recognised cause of summer neuroinvasive disease in the Mediterranean, and the recent documented rise in Italian incidence warrants attention. The clinical essentials are well established, in that exposure is common but disease is not, symptomatic infection is mainly neuroinvasive, most patients recover with supportive care, and a minority are left with meaningful neurological sequelae whose determinants are poorly characterised. The therapeutic position is unchanged, since care is supportive, no licensed antiviral or vaccine exists, and the experimental agents sometimes invoked have no efficacy data and should not be presented as treatment. The preclinical work on peptide therapeutics, RNA interference, and vaccines is worth pursuing but is years from the clinic. In the meantime, the interventions with the greatest near-term value are heightened clinical suspicion in endemic regions, broader access to TOSV-specific molecular diagnostics, integrated human and entomological surveillance, and personal protection against sandfly bites.
